# Editorial: Non-motor Symptoms in Primary Motor Neurological Disorders: From Molecular Pathways to Clinical and Therapeutic Implications

**DOI:** 10.3389/fnins.2019.01296

**Published:** 2019-12-03

**Authors:** Foteini Christidi, Rosa De Micco, Kaylena A. Ehgoetz Martens, Cristina Moglia, Francesca Trojsi

**Affiliations:** ^1^First Department of Neurology, Aeginition Hospital, Medical School, National and Kapodistrian University of Athens, Athens, Greece; ^2^First Division of Neurology, Department of Advanced Medical and Surgical Sciences, University of Campania “Luigi Vanvitelli”, Naples, Italy; ^3^Department of Kinesiology, University of Waterloo, Waterloo, ON, Canada; ^4^ALS Center, “Rita Levi Montalcini” Department of Neuroscience, University of Turin, Turin, Italy

**Keywords:** neurodegenerative diseases, movement disorders, motor neuron disease, non-motor symptoms, *C9orf72* hexanucleotide repeat expansion, neuropsychiatric disorders, sleep disorders, levodopa response

There is increasing evidence that neurodegenerative diseases imply high emotional and economic burden (GBD 2016 Neurology Collaborators, 2019), substantially influenced by a variety of non-motor symptoms (NMS), such as gastrointestinal-, autonomic-, neuropsychiatric-, and sleep disorders, preceding classical motor signs or appearing during the disease course. A convincing explanation of these symptoms would be much better supported by the recently postulated “disease-spreading hypothesis” (Brundin et al., 2010), in that the pathology would not be limited to the initially affected cell populations, but disease would spread involving other non-motor regions, in the brain and beyond (Braak et al., 2003; Brettschneider et al., 2013). The 10 articles contributing to this Frontiers Research Topic will provide readers with an update on some of the most crucial aspects of NMS in neurodegenerative disorders, addressing both molecular and clinical issues.

Firstly, with regard to the use of animal models for investigating the pathogenesis of familial amyotrophic lateral sclerosis (fALS), Pharaoh et al. measured changes in metabolic pathways in spinal cords of the SOD1^G93A^ mouse model of ALS using a targeted proteomic analysis. The protein content of metabolic proteins, including those involved in glycolysis, β-oxidation, and mitochondrial metabolism, was found altered in SOD1^G93A^ mouse spinal cord before disease onset, recalling some hypotheses on the potential role of metabolism and nutrition in ALS pathogenesis (Gallo et al., 2013; Wills et al., 2014).

With regard to clinical studies, assuming the ALS as an emblematic neurodegenerative disease in which both motor and extra-motor symptoms may coexist from early stages, the neuropsychological profile described in ALS patients has been deeply reviewed by Benbrika et al. to provide readers with a clear picture of all the cognitive, emotional and psychological manifestations of ALS. Neuropsychological assessment needs the use of ALS-specific tools, such as the Edinburgh Cognitive and Behavioral ALS screen (ECAS) (Abrahams et al., 2014). Moreover, the increased understanding of the disease spectrum including ALS and frontotemporal dementia (FTD) (Burrell et al., 2016) has led researchers to elaborate on a variety of symptoms not classically considered part of the ALS clinical picture, such as psychiatric symptoms, reviewed by Zucchi et al.. In particular, the link between ALS and schizophrenia was further supported by a substantial genetic correlation, only partially explained by pleiotropic gene variants, such as *C9orf72* (McLaughlin et al., 2017).

ALS patients carrying the *C9orf72* hexanucleotide repeat expansion (C9+ALS) had significantly more co-morbid behavioral variant FTD features than those without (Byrne et al., 2012) and increasing evidence has suggested that hippocampus and subcortical region degeneration also plays a role in the peculiar clinical picture of C9+ALS patients (Agosta et al., 2017). Furthermore, with regard to hippocampal involvement in sporadic ALS, Gómez-Pinedo et al. explored the expression of the intracellular pathway of Notch proteins, probably associated with amyloid precursor protein (APP) signaling pathways (Ables et al., 2011), by analyzing hippocampal samples from the autopsies of 12 patients with ALS or ALS-FTD. They reported lower levels of the Notch intracellular domain in patients with ALS than in controls, leading to increases in amyloid-β and to decrease in hippocampal neurogenesis.

Among the subcortical structures potentially involved in the cognitive status of ALS patients, Consonni et al. tested the relationship between cerebellar degeneration, cognitive syndromes, and *C9orf72* mutation in ALS patients, revealing that cerebellar involvement may reflect a signature of ALS-FTD (Bede et al., 2013) other than a signature of the *C9orf72* hexanucleotide repeat.

In light of the recent observation of a widespread extra-motor involvement in ALS, that has implied that, beyond motor neurons, peripheral axons and nerve terminals may also be affected as early events, Gentile et al. reviewed the most relevant literature regarding common molecular pathways (i.e., impairments of axonal transport, RNA metabolism, and proteostasis) shared by ALS and peripheral axonal neuropathies. They underlined that an extensive evaluation of the molecular events occurring in the peripheral nervous system could be fundamental to understand the pathogenic mechanisms of ALS as well as other neuropsychiatric disorders. In this regard, an interesting link between hereditary polyneuropathies and psychiatric disorders, such as schizophrenia, has been discussed by Endres et al., who presented the case of a patient with schizophrenia and comorbid hereditary neuropathy with liability to pressure palsy (HNPP), due to a deletion of the peripheral myelin protein 22 gene (*PMP22*). This potential association has been explained by the role of *PMP22*, mainly expressed in the peripheral nervous system, although its mRNA has also been detected in the brain (Chanson et al., 2013). In particular, *PMP22* seems to play an important role in regulating cell growth and myelination (Sanahuja et al., 2005), also impaired in schizophrenia.

A widespread appearance of pathologic proteins aggregates in both the central and peripheral nervous systems, including the enteric nervous system (ENS), has been recognized also in Parkinson's disease (PD) (Goedert, 2001). In particular, Fonseca Santos et al. focused on the hypothesis of a “gut-brain axis”: gut toxins could induce the formation of α-synuclein aggregates in the ENS, which may then be transmitted in a prion-like manner to the central nervous system through the vagus nerve. From the therapeutic point of view, levodopa is actually considered the best current symptomatic treatment for PD, although characterizing levodopa response may be a challenge in early stages. In this regard, Serrano et al. presented the analysis of electroencephalography microstates (EEG-MS) default-mode network changes in response to dopaminergic stimulation as a potential tool to evaluate, in a non-invasive way, the levodopa response and to assess the suitability of the patients' medication dosage.

Finally, among other common NMS, Herzog-Krzywoszanska and Krzywoszanski addressed the role of sleep abnormalities in Huntington's disease (HD). Among the major criticisms raised, the authors underlined that many medications administered to HD patients to alleviate motor and psychiatric symptoms may change sleep architecture, thereby negatively impacting sleep quality (Arnulf et al., 2008). Furthermore, more detailed knowledge of these problems in HD can also provide more profound insight into the nature of the neurodegenerative process underlying the disease (Morton, 2013).

In conclusion, the substantial message of this Frontiers Research Topic is that the identification of extra-motor abnormalities may represent a core feature for supporting the diagnosis and predicting the prognosis of many primary motor neurodegenerative disorders and for shedding light on several dysfunctional pathways in order to prompt the development of combined therapies with synergistic neuroprotective effects on several neurodegenerative pathomechanisms.

## Author Contributions

All authors listed have made a substantial, direct and intellectual contribution to the work, and approved it for publication.

### Conflict of Interest

The authors declare that the research was conducted in the absence of any commercial or financial relationships that could be construed as a potential conflict of interest.
